# Case Report: Extraocular muscles paralysis associated with GAD65 antibody: a case series study

**DOI:** 10.3389/fimmu.2023.1256089

**Published:** 2023-12-01

**Authors:** Heyu Zhang, Jiajia Yue, Chun Lian, Youming Long, Dan He

**Affiliations:** ^1^ Department of Neurology, The First Affiliated Hospital, Sun Yat-sen University, Guangdong Provincial Key Laboratory of Diagnosis and Treatment of Major Neurological Diseases, National Key Clinical Department and Key Discipline of Neurology, Guangzhou, China; ^2^ Key Laboratory of Neurogenetics and Channelopathies of Guangdong Province and The Ministry of Education of China, Institute of Neuroscience and The Second Affiliated Hospital of Guangzhou Medical University, Guangzhou, Guangdong, China

**Keywords:** extraocular muscles paralysis, neuro-immune, neuromuscular disorder, autoimmune, GAD65 antibody

## Abstract

**Objective:**

To explore the clinical manifestations of glutamic acid decarboxylase 65 (GAD65) antibody-positive patients with extraocular symptoms and the possible mechanism.

**Method:**

Assays for the presence of GAD65 antibodies were performed on patients’ serum and cerebral spinal fluid (CSF). The brain and ocular structures involved in eye movement were assessed via magnetic resonance imaging (MRI). Tests such as electromyography (EMG), particularly repetitive nerve stimulation (RNS), and neostigmine tests were utilized for differential diagnosis. Additionally, the interaction of GAD65 antibodies with muscle tissue was confirmed using immunofluorescence techniques.

**Result:**

Each patient exhibited symptoms akin to extraocular myasthenia gravis (MG), with two individuals reporting diplopia and two experiencing ptosis. GAD65 antibodies were detected in either the serum or CSF, which were shown to bind with monkey cerebellum slides and mouse muscle slides. Neuroimaging of the brain and extraocular muscles via MRI showed no abnormalities, and all patients tested negative for the neostigmine test, RNS *via* EMG, and the presence of MG antibodies. However, thyroid-related antibodies were found to be abnormal in four of the patients.

**Conclusion:**

Our results showed that GAD65 antibodies are not only associated with encephalitis, cerebellum ataxia or stiff-person syndrome caused by the decrease of GABAergic transmission but also diplopia and ptosis. Therefore, we should pay more attention to extraocular muscle paralysis patients without pathogenic antibodies directed against the components of neuromuscular junctions.

## Introduction

Glutamic acid decarboxylase (GAD) is an enzyme that catalyzes the conversion of the inhibitory neurotransmitter γ-aminobutyric acid (GABA) to glutamate. It is selectively expressed in nerve terminals of presynaptic GABAergic neurons and pancreatic β cells (1). Previous studies reported that Anti-GAD65 antibodies (GAD65-Abs) could be seen in patients with progressive cerebellar ataxia, limbic encephalitis, epilepsy, myelitis, palatal tremor, myoclonus, and even a helpful biomarker of stiff-person syndrome (SPS) (2, 3).

Most of the previous studies have focused on the role of GAD65-Abs interfering in GABAergic synaptic transmission, and some researchers assumed that the inflammatory cascade induced by GAD65-Abs is the cause of neuronal loss and cerebellar atrophy. But there are few studies on its possible role in muscle and neuromuscular junction. Moreover, only 1 case has reported GAD65-Abs and abnormal eye movement (4). We found that some patients with ptosis or diplopia accompanied by thyroid-associated antibodies positive were misdiagnosed as MG and ineffective treatment.

The present study analyzed extraocular muscle activity, antibody titers, and immunofluorescence assays of antibody binding to muscle membranes in four GAD65-Abs positive patients.

## Methods

### Study population

Patients with diagnosed ptosis and eye movement irregularities who tested positive for GAD65-Abs in either serum or CSF were selected for this study. These patients were admitted to either The First Affiliated Hospital of Sun Yat-sen University or The Second Affiliated Hospital of Guangzhou Medical University from January 1, 2022, to December 31, 2022. Prior to inclusion in this research, every patient gave their informed written consent, and the study secured ethical approval.

### Anti-GAD65 antibody assay

Stored at -80°C, serum and CSF samples were assessed for GAD65 antibody presence. The analysis was performed utilizing a commercial kit (Euroimmun, Luebeck, Germany) designed to assay anti-GAD65 antibody, which employs a GAD65-transfected cell line derived from human embryonic kidney 293 cells, as delineated previously (5).

### Immunofluorescence assay for GAD65 antibody

A tissue-based assay confirmed the positive samples of CSF and serum. Briefly, serum (1:10) or CSF(1:50) diluted in phosphate-buffered saline (PBS) was reacted with monkey cerebellum tissue slides provide by IbnSinaHealth (Guangzhou) Technology Co.,Ltd and mouse muscle slides for 3 h at room temperature. The slides were rinsed twice with PBS and then incubated with fluorescein-conjugated goat anti-human IgG for 2 h. Finally, the slides were rinsed with PBS, and the fluorescence intensity was examined under a microscope (6).

## Results

**Figure 1:**
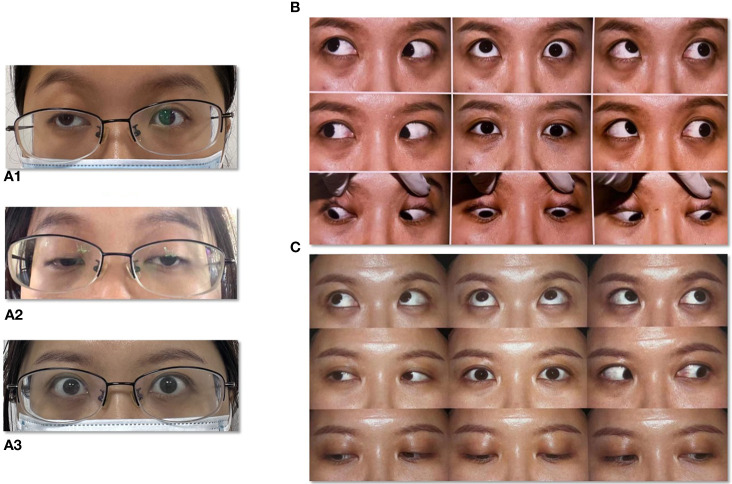
Extraocular paralysis of a typical patient. **(A1)** Right side ptosis at stage of first onset. **(A2)** Bilateral ptosis at stage of second onset. **(A3)** Bilateral eyelids after treatment. **(B)** Eye movement at stage of onset. **(C)** Eye movement after treatment.

During the study period, 14 patients were detected GAD65-Abs positive in serum or CSF. Four of these patients with extraocular symptoms were identified and included in the study. The patient’s neurological symptoms and examination results are summarized in the table. All complained of diplopia, and physical examination showed limited eye movement, vertical or horizontal, without misalignment, and no abnormal pupil diameter or light reflection was found. (**Figure 1**). All patients had an abnormality of thyroid-related antibodies; two (patients 1 and 3) had ptosis; All patients without other symptoms and signs of myopathy. The neostigmine test, RNS of EMG and the antibodies of MG including anti-AchR,anti-Musk and anti-LRP4 were negative in all patients. In order to distinguish brainstem encephalitis and cranial nerve injury associated with GAD65-Abs, brain and orbit MRIs were performed in all patients with diplopia.

**Figure 2:**
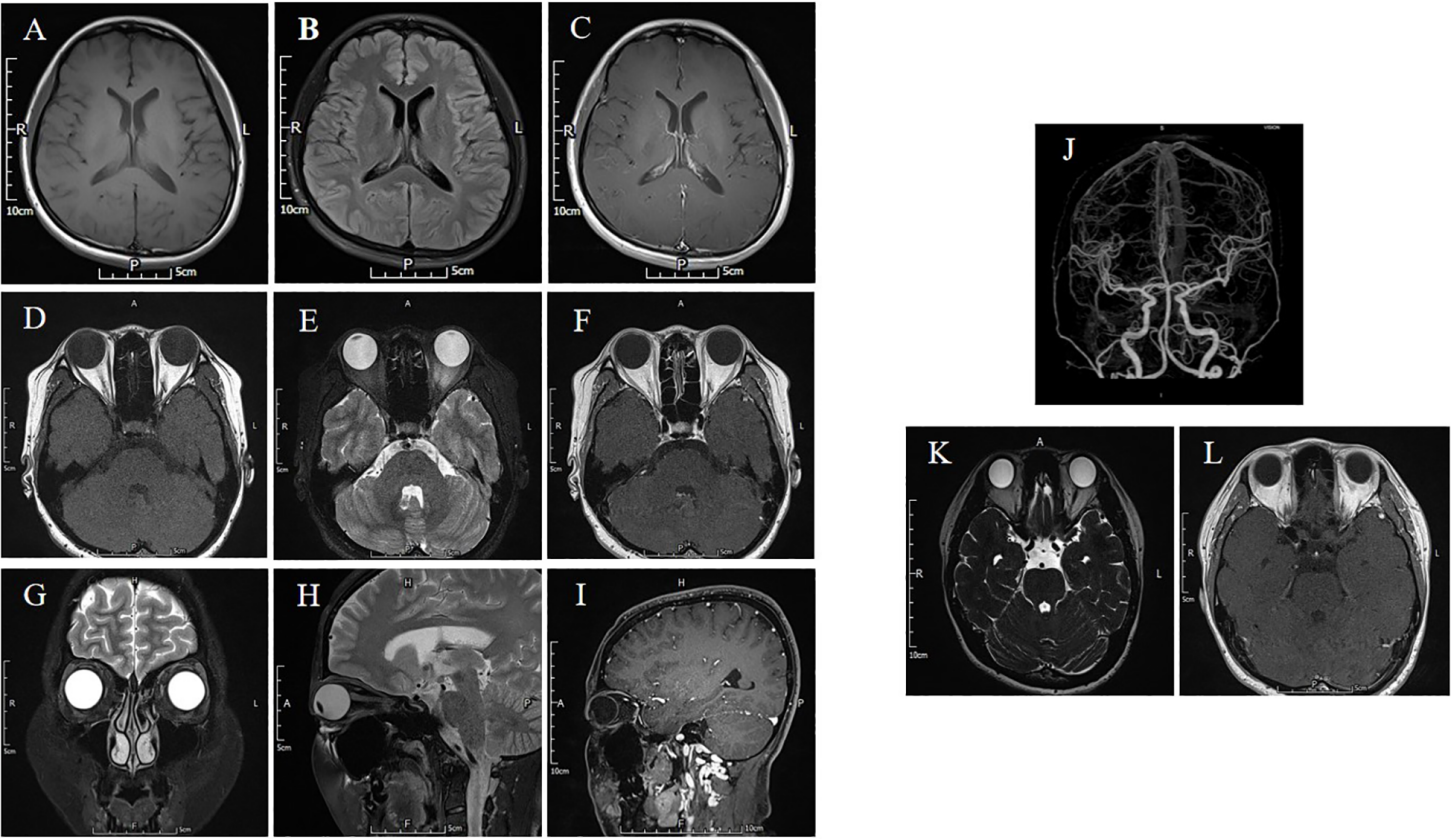
The brain and orbital MRI are normal. **(A-C)** The T1-weighted, T2 FLAIR and T1-weighted contrast-enhanced sequence of barin. **(D-F)** The T1-weighted, T2 FLAIR and T1-weighted contrast-enhanced sequence showed no ocular muscle abnormalities. **(G)** The orbital coronal MRI is normal. **(H, I)** The T2 FLAIR and T1-weighted contrast-enhanced sequence of orbital sagittal MRI. **(J)** The CTA of cranial arteries. **(K, L)** The MRI of oculomotor nerve in T2 Space and T1-weighted contrast-enhanced sequence is normal.

Specifically, patient 1 presented with ptosis and diplopia, with no significant variations observed between morning and evening. MRI scans of the brain, cranial nerve and extraocular muscles were normal (**Figure 2**). Despite negative results for MG-related antibodies and EMG, an initial misdiagnosis of MG was made, but treatment with pyridostigmine bromide, glucocorticoid therapy, and intravenous human immunoglobulin demonstrated poor efficacy. Patient 2 experienced isolated diplopia following a night shift and had a history of leukoderma, which required melanin grafting two years prior. CT and MRI scans conducted for this patient showed no abnormalities. Patient 3 presented with unilateral right-sided ptosis in 2017, with no abnormalities in antibodies of MG, neostigmine test, RNS, brain and orbital MRI. No efficacy of pyridostigmine bromide treatment were observe. But the patient experienced symptom relief within a few months without recurrence even stopped taking medicine. She developed epilepsy in November 2022. Subsequent MRI scans of the brain suggested bilateral hippocampal and adjacent gyrus swelling, suggestive of autoimmune encephalitis. Patient 4 exhibited diplopia with minor ocular discomfort. In addition, she experienced dryness of the eyes and mouth, but sicca syndrome indices were negative, and an MRI scan appeared normal. Interestingly, patients 1, 3, and 4 displayed poor responses to high-dose methylprednisolone treatment but showed marked improvement following therapy with plasma exchange (PE) or immunoadsorption.

**Figure 3:**
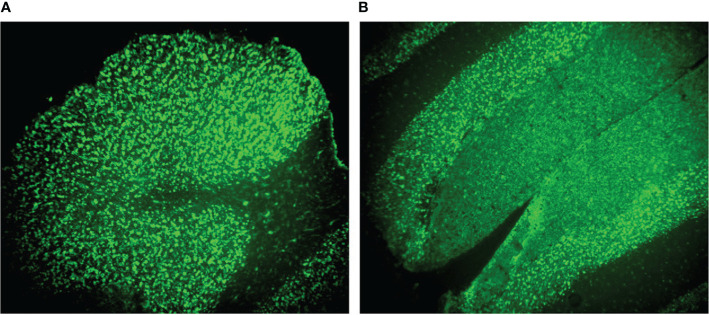
**(A)** A monkey cerebellum slide treated with the typical patient CSF. **(B)** A monkey cerebellum slide treated with the typical patient serum. Obvious staining (green) is observed around granule cells in the granule layer, and weaker staining is present in the molecular layer.

**Figure 4:**
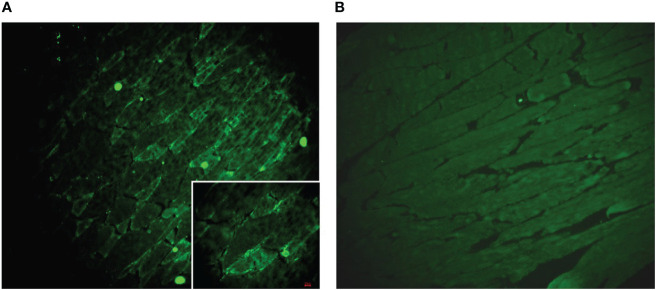
**(A)** A muscle slide treated with typical patient CSF. Obvious staining (green) is observed on the membrane of the myocyte. **(B)** A muscle slide as a negative control.

To explore the possible interaction of GAD65-Abs and diplopia, we incubated the serum and CSF with monkey brain tissue and mouse muscle slides. We found that serum and CSF IgG bound to neurons in the granular layer of the monkey cerebellum, consistent with GAD65 distribution characteristics (**Figure 3**). Furthermore, we found that CSF IgG could bind to the myocyte membrane (**Figure 4**). It demonstrated that GAD65-Abs could attach to myocytes through the directly bind of myocyte or the receptor on the membrane.

To improve the patient’s symptoms, we carried out corresponding treatment. It was found that patients 1 and 3 did not respond well to methylprednisolone pulse therapy and human immunoglobulin pulse therapy, and plasma exchange therapy was effective in reducing GAD65 titer and improving symptoms (**Figure 1**). Patient 2 responded well to a 500mg methylprednisolone pulse. The treatment of patient 4 with corticosteroids and immunosuppressants was not effective. Rituximab can reduce the antibody titer, but symptoms were not significantly relieved.

## Discussion

GAD-Abs are associated with multiple neurological syndromes, including SPS, cerebellar ataxia, nystagmus, palatal myoclonus and limbic encephalitis (5). These syndromes are considered to be caused by the decrease in GABA transmission. The presence of high titers of GAD-Abs in either serum or CSF is regarded as the foundational basis for diagnosis (7). Researchers focused on the function of GAD65-Abs on neuron loss or injury in the central nervous system. However, because of its wide distribution, it can also cause extra-central symptoms, such as type 1 diabetes mellitus (T1DM), thyroiditis, pernicious anemia, coeliac disease, and vitiligo (1). Here we presented the ocular MG-like signs and symptoms which GAD65-Abs may cause. Our study found four patients had MG-like symptoms, which presented ptosis and diplopia with no difference in morning and night; partial eye movement limitation without nystagmus in neurological examination; all had normal brain and orbit MRI, EMG, and MG-associated antibodies negative. Although the clinical symptoms of 4 patients were not severe and were mostly ignored, one patient developed signs of encephalitis after symptoms improved, which suggests that we should make an early diagnosis and implement effective intervention measures. Further, we detected GAD65-Abs in these patients’ serum and CSF, and the GAD65-Abs could bind to mouse muscle, suggesting that GAD65-Abs could go through the blood-brain barrier and might be a novel factor to attack extraocular muscles.

The pathogenicity of GAD-Abs is still controversial. Some studies believe that GAD-Ab is mainly a T cell-mediated immune response (8). In contrast, a few studies have confirmed that intrathecal injection of GAD-Abs from SPS or CA patients is sufficient to reproduce typical disease manifestations in animal models, supporting the hypothesis that GAD-Ab may exhibit a direct pathogenic mechanism (9, 10). Further to this, our study found that CSF GAD65-Abs could cross-react with the membrane of myocytes, suggesting that GAD65-Abs may bind directly to receptors on the myocyte membrane, much like an antigen-antibody reaction, and thus play a important role in regulating movement in the eyelids and extraocular muscles. However, we only verified the combine of GAD65-Abs and myocytes, but whether the GAD65-Abs react with oculo motor nerves should be studied in the future.

GAD65-Abs typically have a personal or familial history of autoimmunity, thyroiditis is common (3). In addition to nervous system diseases, abnormal eye movement in patients requires differentiation from thyroid ophthalmopathy. Therefore, we assessed thyroid-related antibodies and thyroid function in all participants. Notably, three patients tested positive for TPO-Abs and TR-Abs, which indicates the need for cautious differentiation from Graves’ ophthalmopathy. Graves’ ophthalmopathy typically presents restrictive rather than paralytic diplopia due to inflammation and swelling of the extraocular muscles (11, 12). All patients underwent an orbital MRI to evaluate the morphology of their extraocular muscles, with results showing no abnormalities suggestive of Graves’ ophthalmopathy. Many studies have proposed fibroblasts as the target cells in Graves’ ophthalmopathy (13). In response to autoimmune cytokines, fibroblasts secrete large amounts of hyaluronic acid, leading to muscle enlargement and infiltration of adipocytes into the orbit rather than acting directly on the muscle (14, 15). Consequently, we hypothesize that the diplopia observed in these three patients is unrelated to thyroid eye disease.

This study has several limitations. Primarily, the small sample size and the specific focus on the dysfunction of eye muscles without a broader investigation of the involvement of other skeletal muscles. We observed that patients with GAD65 antibody positive in current case have isolated clinical characteristics of extraocular muscle dysfunction, suggesting that in clinical diagnosis and treatment, attention should be paid to identifying whether there is abnormal expression of GAD65 in patients with unknown extraocular muscle dysfunction. However, it is not clear whether GAD65 antibody plays a major role in the pathogenic mechanism of these patients, and it is still necessary to further study and clarify the relevant mechanism of interaction between GAD65 antibody and cranial nerve or extraocular muscle. Our findings serve as a springboard for future research into the cellular and molecular mechanisms of GAD65-Abs in neurological syndromes.

## Data availability statement

The raw data supporting the conclusions of this article will be made available by the authors, without undue reservation.

## Ethics statement

The studies involving humans were approved by The First Affiliated Hospital of Sun Yat-sen University ethics committee. The studies were conducted in accordance with the local legislation and institutional requirements. The participants provided their written informed consent to participate in this study. The study was conducted in accordance with the local legislation and institutional requirements. Written informed consent was obtained from the individual(s) for the publication of any potentially identifiable images or data included in this article.

## Author contributions

HZ: Data curation, Formal Analysis, Funding acquisition, Resources, Writing – original draft, Writing – review & editing. JY: Data curation, Investigation, Methodology, Writing – review & editing. CL: Investigation, Methodology, Writing – review & editing. YL: Conceptualization, Methodology, Supervision, Validation, Writing – review & editing. DH: Conceptualization, Funding acquisition, Project administration, Supervision, Validation, Writing – review & editing.
