# Supplementary material for: Case Report: Extraocular muscles paralysis associated with GAD65 antibody: a case series study
Source: Front Immunol. 2023 Dec 1;14:1256089. doi: 10.3389/fimmu.2023.1256089 (PMC10722167; doi:10.3389/fimmu.2023.1256089)
Supplement: Supplementary file 1 [file Table_1.docx]

| Patient | first onset | second onset | third onset |
| --- | --- | --- | --- |
| 1 | left eye upward limitation  left ptosis | bilateral ptosis，left eye upward，adduction and abduction limitation | right eye upward and abduction limitation |
| 2 | right eye downward and lateral lower limitation |  |  |
| 3 | right ptosis，without diplopia |  |  |
| 4 | left eye downward limitation |  |  |

Supplement Table 1 The neurological examination of patients
